# Burden of pathogenic variants in genes associated with medically actionable disease in the Slovenian population

**DOI:** 10.3389/fgene.2026.1847596

**Published:** 2026-10-01

**Authors:** Urška Kotnik, Luca Lovrecic, Borut Peterlin

**Affiliations:** 1 Clinical Institute of Genomic Medicine, University Medical Centre Ljubljana, Ljubljana, Slovenia; 2 Faculty of Medicine, University of Ljubljana, Ljubljana, Slovenia

**Keywords:** cardiovascular disease, hereditary cancer, medically actionable disease, personalised medicine, population burden

## Abstract

**Introduction:**

The prevalence of pathogenic variants in disease-related genes is population-specific and is of vital importance for the development of national health policies. It is especially important with medically actionable diseases, where early interventions and preventive surveillance are available and can enable effective preventive measures or timely treatment.

**Materials and Methods:**

We have examined the burden of pathogenic variants in 84 genes associated with medically actionable disease in exome sequencing data of 14.000 Slovenian individuals, representing 0.7% of the whole Slovenian population. The variants were classified according to the ACMG criteria, and the prevalence of pathogenic variants in each gene was compared to the burden of pathogenic variants in the gnomAD database.

**Results:**

The prevalence of pathogenic variants in 84 medically actionable genes in the Slovenian population is 2.73%. Furthermore, we identified an additional 0.48% of novel candidate likely pathogenic variants. Together, these variants accounted for 3.21% of all detected potentially medically actionable variants. Of this variation, 1.73% is cardio-related, 1.21% is cancer-related, and 0.27% is associated with other phenotypes. The prevalence of pathogenic variants in *BRCA1, LDLR,* and *HFE* differs significantly between the Slovenian population and the gnomAD database.

**Discussion:**

Revealing the prevalence of pathogenic variants in medically actionable genes in the Slovenian population highlights the need for careful interpretation of genomic data in our population, as approximately one in 31 individuals carries a potentially medically actionable variant. Our results advocate for population-specific evaluation of genetic disease burden, as tailored measures, particularly for genes enriched in a given population, may be warranted.

## 1 Introduction

Next-generation sequencing (NGS) is a powerful genomic technology used for the diagnosis of genetic disorders, personalization of cancer treatment, preimplantation and preconception analysis, among other applications, leading to the development of precision medicine based on genomic insights. The process of NGS results in the generation of a large amount of data (Van Hout et al., 2020; Auton et al., 2015), meaning that genomic diagnosis can frequently reveal additional findings in genes unrelated to the original diagnosis within the patient’s genome. These include incidental findings, which are discovered unintentionally, and secondary findings, which are deliberately sought during the interpretation process (Saelaert et al., 2018). Some of those variants are medically actionable, meaning they are causative for disease for which screening is possible, early diagnosis is beneficial, and preventive measures and/or effective treatment exist. It is therefore in the patient’s interest to return such findings to the patient (Green et al., 2013).

To clarify which genes lead to medically actionable disease, the American College of Medical Genetics and Genomics (ACMG) has compiled, periodically validated, and updated a list of genes associated with highly penetrant diseases for which a well-established intervention or surveillance is available. The first iteration, published in 2013, contained 56 genes, related to 24 different diseases (Green et al., 2013) and was firstly updated in 2017 (2.0 version) (Kalia et al., 2017), where one gene (*MYLK*) was removed and four new genes (*ATP7B*, *BMPR1A*, *SMAD4*, and *OTC*) were added to the list. More importantly, while the first version of the guidelines used the terms incidental and secondary findings interchangeably, the second edition of the guidelines switched the terminology towards secondary findings, since the variants in the genes are intentionally being analysed, as opposed to being found incidentally. The list was then further updated in 2021 (3.0 version), where no genes were removed, but 14 new were added (Miller et al., 2021). After that, the goal has been to update the list annually, with the actual updates being released in 2022 (5 new genes added) (Miller et al., 2022), 2023 (3 genes added) (Miller et al., 2023), and most recently in 2025 (3 genes added) (Lee et al., 2025) (versions 3.1–3.3). The current 33 version contains 84 genes, connected to 39 distinct genetic diseases. The ACMG recommends that the list be used for reporting of secondary findings in a clinical setting, upon genetic counselling and informed consent (ACMG Board of Directors, 2019).

Since the first publication of the ACMG recommendations in 2013 (Green et al., 2013) clinical laboratories offering NGS have begun reporting medically actionable secondary and incidental findings to patients, who opt in. Multiple studies, examining different large unaffected and control populations, indicate that pathogenic variants in the genes of the ACMG list are present in 2%–6% of the population (Halldorsson et al., 2022; Saeidian et al., 2024; Huang et al., 2022; Venner et al., 2024; Gordon et al., 2020), noting that this proportion could have a significant effect on societal disease burden in the studied populations.

Many earlier studies concentrated on multi-ethnic and multi-population cohorts, highlighting the importance of including human diversity in the context of human genomic studies (Saeidian et al., 2024; Gordon et al., 2020; Amendola et al., 2015). Importantly, the proportion of pathogenic variants in genes related to genetic disease varies significantly in human populations (Dorling et al., 2021; Cong et al., 2022), therefore, population-specific results are needed to help assess genomic risks and disease burden in the given population and aid in public health genomic development (Molster et al., 2018). The burden of pathogenic variants has previously rarely been reported in a population-based setting.

We recently established the Slovenian genomic database and browser (Maver et al., 2025), the first large publicly available Central European population genomic resource, enabling us to seamlessly discover the burden of secondary findings in the Slovenian population. Having previously reported on the prevalence of pathogenic variants in the gynecologic cancer predisposition genes and autosomal recessive genes in the Slovenian population (Maver et al., 2025; Kotnik et al., 2023), we were able to estimate the burden of pathogenic variants in genes associated with specific phenotypes and properties of the Slovenian population. This emphasizes the importance of population-based research for understanding the burden of secondary findings.

Here, we present the burden of pathogenic findings in all 84 genes of the current ACMG list version 3.3 (Lee et al., 2025) in 14,000 individuals of Slovenian origin. Our aim was to determine the prevalence of pathogenic variants in genes associated with medically actionable disease in the Slovenian population as a first step towards population-specific evaluation of genetic disease burden and development of as tailored measures.

## 2 Materials and methods

### 2.1 Study population

We have systematically investigated variants in the 84 genes of the ACMG gene list version 3.3 (Lee et al., 2025) in 14,000 individuals who were referred to the Clinical Institute of Genomic Medicine, University Medical Centre, Ljubljana, Slovenia, from July 2014 to March 2025 for exome sequencing for various rare genetic conditions. The participants were referred for variety of different conditions and were distributed across all age groups, including children.

All patients received pre-test genetic counselling by a genetic specialist where the genetic testing, the process of analysis and, possible findings were clarified. The possibility of discovering incidental findings was explained. The participants could opt-in to receive pathogenic and likely pathogenic variants in genes connected to actionable disease. No genetic variants, connected with adult onset disease, were reported to children. The patients or patient’s parents/legal guardians signed an informed consent. Then, for each patient, exome sequencing was performed and the genomic data were analyzed in the context of the clinical indication for testing. Variants classified as pathogenic or likely pathogenic and associated with the referral diagnosis were designated as primary findings and excluded from the analysis. All of the other variants in the patient’s exome were stored in a database. This database of the variants, detected in the exomes of 14,000 patients, unrelated to their diagnosis, presents an approximation of general, unaffected population, as it presents the genetic variability of 14.000 Slovenians, excluding their primary disease-causing variants associated with the patients’ referral indications (Kotnik et al., 2023). GnomAD 4.1 population was chosen as a control database for our study (N = 807,162) except for the *TTN* gene, where gnomAD 3.2.1 was used (N = 76,156) (Karczewski et al., 2020).

### 2.2 Ethical approval and declaration

The patient’s data were non-reciprocally de-identified, and aggregated data on variant frequencies were assessed. All patients gave informed consent for participation. The consent for minors was provided by their patents/legal guardians. All methods were performed in accordance with the Declaration of Helsinki. The study was approved by the Medical Ethics Committee of the Republic of Slovenia (number: 0120-527/2025-2711-7).

### 2.3 Sequencing and variant calling

The samples for exome sequencing were enriched using TruSight One, TruSight Exome, and Nextera Coding Exome capture kits by Illumina or Agilent SureSelect Human All Exon v2 and Agilent SureSelect Human All Exon v5 capture kits by Agilent Technologies. Sequencing was performed on Illumina MiSeq or Illumina HiSeq 2500 platforms. A minimum median exome coverage was 60x, with over 95% of targets covered with at least 10×sequencing depth. Raw sequence files were processed using a custom exome analysis pipeline and aligned to UCSC hg19 human reference genome as previously described by our group (Bergant et al., 2021). We also performed quality control on the individual variants with required variant quality over 20 and variant coverage over 10 for inclusion in further analysis (Bergant et al., 2026).

### 2.4 Gene panel

The 84 genes of the ACMG Secondary Findings v3.3 list (Lee et al., 2025) (Supplementary Table S1). The MANE transcript was used for all genes.

### 2.5 Variant filtering and classification

We have established a rigorous filtering process to extract all the previously described pathogenic variants as well as novel, candidate pathogenic variants, from the variation present in 84 genes in the cohort of 14,000 Slovenian individuals.

The filtering of the newly created database was two-fold (Figure 1). First, we considered all the previously reported (likely) pathogenic variants in ClinVar.

**FIGURE 1:**
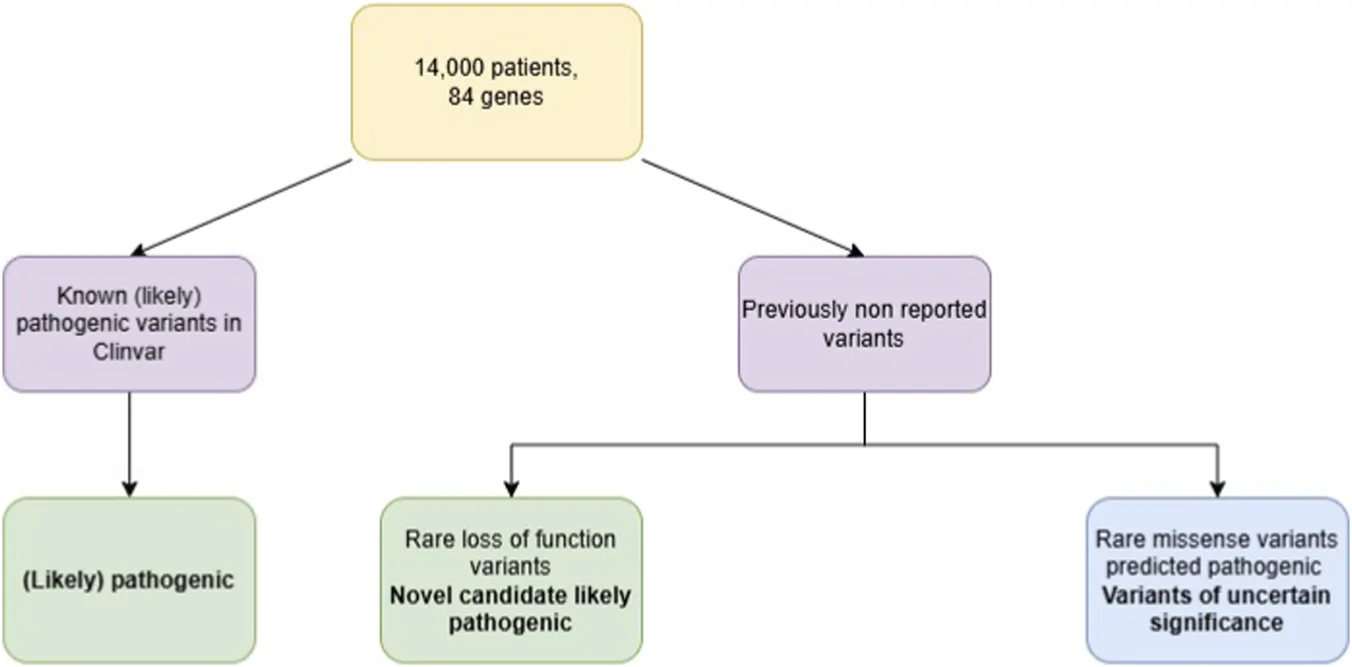
Filtering and classification process.

Secondly, candidate pathogenic variants were examined. Loss-of-function variants in genes, where loss of function is the mechanism of disease, presumably leading to nonsense-mediated decay (not present in the last or the last 50 nucleotide of the penultimate exon), that were present fewer times than the most frequent known pathogenic variant in a gene were considered. Missense variants in genes, where missense is a common mechanism of disease (z-score >3.09), were examined next. The variants previously reported as (likely) benign were excluded. Extremely rare (global frequency <5) variants were further filtered according to Alpha missense (Cheng et al., 2023) predictions; variants where the Alpha missense score was >0.9 were selected. All of the candidate LOF and missense variants were classified according to the ACMG criteria (Richards et al., 2015). PVS1 criterion was applied only for the loss of function variants and was used according to the guidelines recommended by Abou Tayoun et al. (2018). Consequently, LOF variants were classified as likely pathogenic (PVS1, PM2) according to the guidelines. Still, since they were previously unreported in patients, we have decided to refer to the novel LOF variants as novel candidate likely pathogenic variants rather than likely pathogenic variants. Missense variants were all classified as variants of uncertain significance (VUS) (PM2, PP2, PP3).

Several important factors were considered in interpreting the pathogenicity of variants:

1. Mechanism of pathogenicity of specific genes. Only missense variants were considered in: *MYH7*, *TNNI3*, *TPM1*, *PRKAG2*, *MYL2*, *RET*, *RYR1*; only loss-of-function variants in the A band and I band in exons with high pext score were considered in the *TTN* gene. Both types of variants were considered in other genes.
2. Mode of inheritance was considered in accordance with ACMG recommendations (Supplementary Table S1). In X-linked genes (*GLA*, *OTC* and *ABCD1*), all variants were considered. In AR-related genes (*MUTYH*, *CASQ2*, *TRDN*, *BTD*, *GAA*, *RPE65*, *ATP7B, CYP27A1*) only variants in the homozygous state were considered, and in *HFE*, only p.C282Y homozygotes were considered.

### 2.6 Statistical analysis

Finally, we have performed a comparison of the prevalence of (likely) pathogenic variants in our database and of (likely) pathogenic variants in the gnomAD database. For each gene the proportions of pathogenic variants in each of the populations was calculated. The differences in carrier frequencies between cohorts were evaluated using Fisher’s exact test. The magnitude of association between the burden of pathogenic variants in the study and control populations were calculated using the odds ratio 95% confidence interval. The null cells were calculated with the Haldane–Anscombe correction.To account for multiple testing across all analyzed genes, p-values we corrected using the Benjamini–Hochberg false discovery rate procedure. Both unadjusted p-values and FDR-adjusted q-values are reported (Table 1). All statistical analyses were performed using two-sided tests, and <0.01 was considered statistically significant.

**TABLE 1:** Prevalence of (novel candidate likely) pathogenic variants in 84 medically actionable genes in the Slovenian population.

Gene	N PLP Slo	N PCLP Slo	N PLP and NCLP Slo	%PLP Slo	N PLP gnomAD	% PLP gnomAD	OR (95% CI)	p	​	N VUS Slo	% VUS Slo
Genes, related to cancer											
*APC*	3	1	4	0.03%	299	0.04%	0.77 (0.29–2.07)	0.824	1.000	​	​
*RET*	12	​	12	0.09%	347	0.04%	1.99 (1.12–3.55)	0.024	0.312	​	​
*BRCA1*	52	​	52	0.37%	818	0.10%	3.68 (2.78–4.87)	0.000	0.000	​	​
*BRCA2*	41	1	42	0.30%	2898	0.36%	0.84 (0.62–1.13)	0.284	0.809	​	​
*PALB2*	19	​	19	0.14%	720	0.09%	1.52 (0.96–2.40)	0.085	0.603	​	​
*SDHD*	1	​	1	0.01%	187	0.02%	0.31 (0.04–2.19)	0.387	0.910	​	​
*SDHAF2*	1	​	1	0.01%	102	0.01%	0.57 (0.08–4.06)	1.000	1.000	​	​
*SDHC*	​	​	0	0.00%	20	0.00%	1.41 (0.09–22.9)	1.000	1.000	​	​
*SDHB*	3	​	3	0.02%	142	0.02%	1.22 (0.39–3.83)	0.740	1.000	​	​
*MAX*	​	​	0	0.00%	26	0.00%	1.09 (0.07–17.9)	1.000	1.000	​	​
*TMEM127*	1	​	1	0.01%	86	0.01%	0.67 (0.09–4.79)	1.000	1.000	​	​
*BMPR1A*	​	​	0	0.00%	11	0.00%	2.51 (0.15–41.8)	1.000	1.000	3	0.02%
*SMAD4*	1	​	1	0.01%	37	0.00%	1.56 (0.22–11.1)	0.480	1.000	5	0.04%
*TP53*	​	​	0	0.00%	124	0.02%	0.23 (0.01–3.78)	0.281	0.809	​	​
*MLH1*	2	​	2	0.01%	144	0.02%	0.80 (0.20–3.23)	1.000	1.000	​	​
*MSH2*	2	​	2	0.01%	168	0.02%	0.69 (0.17–2.78)	1.000	1.000	​	​
*MSH6*	10	1	11	0.08%	625	0.08%	1.01 (0.55–1.84)	0.878	1.000	​	​
*PMS2*	11	​	11	0.08%	189	0.02%	3.36 (1.84–6.14)	0.001	0.014	​	​
*MEN1*	1	2	3	0.02%	129	0.02%	1.34 (0.43–4.19)	0.495	1.000	7	0.05%
*MUTYH*	​	​	0	0.00%	3	0.00%	8.24 (0.42–162)	1.000	1.000	​	​
*NF2*	1	​	1	0.01%	16	0.00%	3.60 (0.49–26.3)	0.253	0.809	​	​
*STK11*	​	​	0	0.00%	28	0.00%	1.01 (0.06–16.6)	1.000	1.000	​	​
*PTEN*	​	​	0	0.00%	151	0.02%	0.19 (0.01–3.11)	0.192	0.771	2	0.01%
*RB1*	1	​	1	0.01%	69	0.01%	0.84 (0.12–6.01)	1.000	1.000	​	​
*TSC1*	​	​	0	0.00%	20	0.00%	1.41 (0.09–22.9)	1.000	1.000	4	0.03%
*TSC2*	​	​	0	0.00%	128	0.02%	0.22 (0.01–3.62)	0.286	0.809	​	​
*VHL*	​	​	0	0.00%	56	0.01%	0.51 (0.03–8.38)	1.000	1.000	​	​
*WT1*	2	​	2	0.01%	54	0.01%	2.14 (0.53–8.65)	0.247	0.809	​	​
Genes, related to cardiac phenotypes											
*FBN1*	​	1	1	0.01%	190	0.02%	0.30 (0.04–2.15)	0.390	0.910	12	0.09%
*TGFBR1*	​	​	0	0.00%	68	0.01%	0.42 (0.03–6.94)	0.634	1.000	2	0.01%
*TGFBR2*	​	2	2	0.01%	78	0.01%	1.48 (0.36–6.02)	0.397	0.910	​	​
*SMAD3*	​	​	0	0.00%	53	0.01%	0.54 (0.03–8.85)	1.000	1.000	3	0.02%
*ACTA2*	1	​	1	0.01%	51	0.01%	1.13 (0.16–8.08)	0.591	1.000	4	0.03%
*MYH11*	1	​	1	0.02%	232	0.03%	0.25 (0.04–1.79)	0.198	0.771	18	0.13%
*PKP2*	1	​	1	0.01%	250	0.03%	0.23 (0.03–1.66)	0.139	0.682	​	​
*DSP*	1	5	6	0.04%	419	0.05%	0.83 (0.37–1.86)	0.850	1.000	​	​
*DSC2*	​	3	3	0.02%	202	0.03%	0.86 (0.27–2.70)	1.000	1.000	​	​
*TMEM43*	2	​	2	0.01%	155	0.02%	0.74 (0.18–2.98)	1.000	1.000	​	​
*DSG2*	9	​	9	0.06%	283	0.04%	1.83 (0.93–3.60)	0.105	0.651	​	​
*RYR2*	1	​	1	0.01%	26	0.00%	2.22 (0.31–15.9)	0.371	0.910	20	0.14%
*CASQ2*	​	​	0	0.00%	1	0.00%	19.2 (0.78–472)	1.000	1.000	​	​
*TRDN*	​	​	0	0.00%	0	0.00%	NA	1.000	1.000	​	​
*TNNT2*	​	​	0	0.00%	105	0.01%	0.27 (0.02–4.48)	0.431	0.960	​	​
*LMNA*	​	​	0	0.00%	156	0.02%	0.18 (0.01–3.03)	0.120	0.670	4	0.03%
*FLNC*	4	​	4	0.03%	209	0.03%	1.10 (0.41–2.95)	0.787	1.000	21	0.15%
*TTN*	24	40	64	0.46%	339	0.45%	1.03 (0.79–1.34)	0.836	1.000	​	​
*BAG3*	​	3	3	0.02%	57	0.01%	3.03 (0.95–9.71)	0.083	0.603	​	​
*DES*	1	​	1	0.01%	109	0.01%	0.53 (0.07–3.81)	1.000	1.000	​	​
*RBM20*	1	1	2	0.01%	287	0.04%	0.40 (0.10–1.60)	0.253	0.809	​	​
*TNNC1*	​	​	0	0.00%	58	0.01%	0.49 (0.03–8.06)	0.629	1.000	​	​
*COL3A1*	​	1	1	0.01%	162	0.02%	0.36 (0.05–2.57)	0.535	1.000	3	0.02%
*LDLR*	72	​	72	0.51%	2622	0.32%	1.58 (1.25–2.01)	0.000	0.008	​	​
*APOB*	12	​	12	0.09%	796	0.10%	0.87 (0.49–1.54)	0.785	1.000	​	​
*PCSK9*	6	​	6	0.04%	780	0.10%	0.44 (0.20–0.98)	0.038	0.368	​	​
*MYH7*	6	1	7	0.05%	158	0.02%	2.55 (1.16–5.58)	0.024	0.312	3	0.02%
*MYBPC3*	20	​	20	0.14%	1661	0.21%	0.69 (0.44–1.08)	0.109	0.651	​	​
*TNNI3*	2	​	2	0.01%	38	0.00%	3.03 (0.75–12.3)	0.149	0.682	​	​
*TPM1*	​	​	0	0.00%	16	0.00%	1.76 (0.10–30.0)	1.000	1.000	​	​
*MYL3*	​	​	0	0.00%	65	0.01%	0.44 (0.03–7.17)	0.631	1.000	​	​
*ACTC1*	​	​	0	0.00%	52	0.01%	0.54 (0.03–8.93)	1.000	1.000	​	​
*PRKAG2*	​	​	0	0.00%	2	0.00%	9.62 (0.39–237)	1.000	1.000	​	​
*MYL2*	1	​	1	0.01%	8	0.00%	7.21 (0.90–57.9)	0.143	0.682	​	​
*KCNQ1*	9	2	11	0.08%	471	0.06%	1.35 (0.73–2.49)	0.291	0.809	​	​
*KCNH2*	1	1	2	0.01%	274	0.03%	0.42 (0.10–1.70)	0.344	0.910	​	​
*SCN5A*	5	1	6	0.04%	349	0.04%	0.99 (0.44–2.22)	1.000	1.000	14	0.1%
*CALM1*	​	​	0	0.00%	2	0.00%	9.62 (0.39–237)	1.000	1.000	​	​
*CALM2*	​	​	0	0.00%	6	0.00%	3.21 (0.17–61.5)	1.000	1.000	​	​
*CALM3*	​	​	0	0.00%	10	0.00%	2.02 (0.12–34.5)	1.000	1.000	​	​
*PLN*	1	​	1	0.01%	9	0.00%	6.41 (0.80–51.4)	0.158	0.685	​	​
Genes, related to other phenotypes											
*HFE*	18	​	18	0.13%	3399	0.42%	0.31 (020–0.49)	0.000	0.000	​	​
*ACVRL1*	**1**	​	1	0.01%	49	0.01%	1.18 (0.17–8.44)	0.577	1.000	4	0.03%
*ENG*	​	​	0	0.00%	113	0.01%	0.25 (0.02–4.08)	0.271	0.809	​	​
*RYR1*	​	​	0	0.00%	221	0.03%	0.13(0.0081–2.09)	0.037	0.368	79	0.56%
*CACNA1S*	4	1	5	0.04%	370	0.05%	0.78 (0.32–1.93)	0.840	1.000	​	​
*HNF1A*	13	​	13	0.09%	449	0.06%	1.67 (0.95–2.94)1.67	0.071	0.603	​	​
*RPE65*	​	​	0	0.00%	0	0.00%	NA	1.000	1.000	​	​
*ATP7B*	​	​	0	0.00%	0	0.00%	NA	1.000	1.000	​	​
*TTR*	1	​	1	0.01%	28	0.00%	2.06 (0.29–14.7)	0.393	0.910	​	​
***Cancer***	​	​	169	1.21%	7659	0.94%	​	​	​	​	​
***Cardiac***	​	​	242	1.73%	9113	1.50%	​	​	​	​	​
***Other***	​	​	38	0.27%	5146	0.65%	​	​	​	​	​
***Sum***	​	​	449	3.21%	21921	3.09%	​	​	​	​	​

Legend: SLO: 14.000 individuals of the Slovenian population, VUS: variant of uncertain significance, PLP: pathogenic, likely pathogenic, NCLP: novel candidate likely pathogenic. Bold values are statistically significant. Genes, connected to the inborn errors of metabolism (*BTD*, *GLA*, *OTC*, *GAA*, *CYP27A1*, *ABCD1*), are not included in the table, since no carriers of pathogenic variants in those genes have been found.

## 3 Results

After rigorous manual classification, the study has revealed the presence of 449 (novel candidate likely) pathogenic variants in 50 out of 84 genes in our cohort, resulting in a cumulative prevalence of 3.21%: 2.73% of those variation present pathogenic variants, that were previously reported in ClinVar, and 0.48% of those variants were novel candidate likely pathogenic variants. All of the variants, except in the *HFE* gene, were found in heterozygous state. This variation is presented in Table 1. The full list of variants in Supplementary Table S2.

In the Slovenian population, the pathogenic variants and novel candidate likely pathogenic variants in medically actionable genes were most frequently found in cardiac disease-related genes (1.73%) and hereditary cancer-related genes (1.21%), which represent the majority of the list; the remaining 0.27% were found in genes related to other phenotypes. No variants were found in genes related to inborn errors of metabolism. This suggests that one individual in 31 in the Slovenian population harbours a (likely) pathogenic or a novel candidate likely pathogenic variant in a medically relevant gene. Pathogenic variants in those same genes are present in 3.10% of the gnomAD population.

Considering genes associated with cardiovascular phenotypes, variants in two genes are very frequent in the Slovenian population. The highest number of pathogenic variants is found in *LDLR* (0.51%), resulting in a strong burden of predisposition to hypercholesterolemia in the Slovenian population. Comparison with gnomAD revealed that pathogenic variants are significantly more frequently present in the Slovenian population compared with the gnomAD population in the *LDLR* gene (p < 0.01) as well. The second gene, where a high number of (novel candidate likely) pathogenic findings is present, is *TTN,* as 0.46% of the cohort are harbouring a pathogenic loss-of-function variant in this cardiomyopathy predisposition-related gene. Pathogenic variants in *MYBPC3* are present in 0.14% of the population, and in other cardiac disease-related genes were present in 0.09%–0.01% of the cohort. No pathogenic or novel candidate likely pathogenic variants have been found in *TGFBR1*, *SMAD3*, *CASQ2*, *TRDN*, *TNNT2*, *TPM1*, *MYL3*, *ACTC1*, *PRKAG2*, *CALM1*, *CALM2*, and *CALM3* genes in the Slovenian population.

Among the cancer-related genes, hereditary breast and ovarian cancer-related genes, namely, *BRCA1*, *BRCA2*, and *PALB2*, show the highest proportion of pathogenic or novel candidate likely pathogenic variants, with 0.37%, 0.30%, and 0.14%, respectively. Pathogenic variants in *BRCA1* are significantly more frequently present in the Slovenian population than in the gnomAD population (0.37% vs. 0.1%, p < 0.01). When considering other hereditary cancer syndromes, (novel candidate likely) pathogenic variants in MMR genes (*MLH1*, *MSH2*, *MSH6*, and *PMS2*), causative for Lynch syndrome, are most frequent, as they are cumulatively present in 0.18% of the population, which has an increased risk of developing non-polyposis colorectal cancer. Pathogenic and novel candidate likely pathogenic variants in multiple endocrine neoplasia genes are present in 0.11% of the population (*RET* 0.09% and *MEN1* 0.02%), and pathogenic variants in succinate dehydrogenase genes (*SDHAF2*, *SDHB*, *SDHD*) are cumulatively present in 0.03% of the studied population. Pathogenic and novel candidate likely pathogenic variants in *APC* are present in 0.03% of the population. In other genes, pathogenic variation is less frequent: 0.01% in each *SMAD4*, *NF2*, *RB1*, *TMEM127,* and *WT1*. No pathogenic variants or novel candidate likely pathogenic variants have been discovered in *SDHC*, *MAX*, *BMPR1A*, *TP53*, *MUTYH*, *STK11*, *PTEN*, *TSC1*, *TSC2*, and *VHL*.

No pathogenic or novel candidate likely pathogenic variants were identified in genes associated with inborn errors of metabolism (*BTD*, *GLA*, *OTC*, *GAA, CYP27A1, ABCD1*) in our cohort. Amongst the genes related to miscellaneous phenotypes, the highest number of pathogenic variants (0.13%) is found in *HFE*, associated with hemochromatosis. This represents the number of homozygotes carrying the *HFE* p.C282Y variant in the studied population. However, this number is significantly lower than the prevalence in the gnomAD database (p < 0.01). Secondly, 0.09% of individuals harbour a pathogenic variant in the *HNF1A* gene related to diabetes, 0.4% in *CACNA1S*, related to malignant hyperthermia (one of those individuals carries a novel candidate likely pathogenic variant), 0.01% in *ACVRL1*, related to hereditary haemorrhagic telangiectasia, and 0.01% in TTR, related to amyloidosis. No pathogenic or novel candidate likely pathogenic variants were found in *ENG*, *RPE65*, *RYR1,* and *ATP7B*.

We have additionally discovered 208 interesting VUS variants, present in 1.49% of the cohort (Table 1; Supplementary Table S2).

## 4 Discussion

The burden of genetic findings, including the prevalence of pathogenic variants in medically actionable disease, is population-specific (Jiang and Assis, 2020). Therefore, it is crucial to determine the rate of such findings in a population to develop health policies, such as appropriate specific screening and surveillance protocols, as well as identify diseases of interest in a particular population ((Molster et al., 2018)). The aim of our study was to determine the burden of the pathogenic variants in medically actionable genes in the Slovenian population as a first step informing the development of population-specific health policies. We have examined the prevalence of (likely) pathogenic genomic variants in serious disease, associated with medically actionable genes in the Slovenian population, and discovered a 2.73% burden of (likely) pathogenic variants; 0.48% of this variation was novel, presenting novel candidate likely pathogenic variants. Additionally, we have detected 1.49% of interesting VUS variants. This estimate presents the first report of the population burden of pathogenic findings in medically actionable genes connected to serious disease in the Slovenian population and one of the first to show this burden on a population level, and as such, warrants consideration in both scientific research and clinical practice.

The cardiac disease-related genes present the largest group amongst the medicinally actionable genes, and pathogenic and novel candidate likely pathogenic variants in those genes were most frequently found in our study (in 1.73% of the cohort). A recent study, classifying the genetic variation in Slovenian patients with cardiomyopathies, has revealed that the highest prevalence of pathogenic variants in the Slovenian population is discovered in the *TTN* gene, which is the second most frequently affected gene in our study as well. In the Slovenian patients, this gene is followed by *MYBPC3*, *MYH7*, and *DSP*, all of which have a prominent prevalence in our study (Vodnjov, 2025). Our study has shown that the Slovenian population is enriched by pathogenic variants in the *LDLR* gene, causative for familial hypercholesterolemia. Slovenia has a universal screening program for children for familial hypercholesterolemia, based on detecting elevated levels of cholesterol, followed by genetic testing, which has revealed that the prevalence of genetically confirmed disease is one in 431 in Slovenian 5-year-olds (0.23%) (Sustar et al., 2022). The higher prevalence (0.51%) reported in our cohort is possibly due to performing genetic testing only in children with elevated cholesterol levels and not population-wide; patients exhibiting later onset of the disease and variants leading to milder effects may be missed in the metabolic screening program.

Pathogenic and novel candidate likely pathogenic variants were found in 1.21% the population in hereditary cancer-related genes. We have previously reported a prevalence of pathogenic variants in a different, broader panel of hereditary breast and gynecological cancer-related genes in the Slovenian population, the prevalence amongst the genes, included in this study was 2.14% in the population of 7091, and the highest prevalence amongst the genes from our list was described in *BRCA1*, *BRCA2,* and *PALB2* genes; higher prevalence of *BRCA1* pathogenic variants compared to the gnomAD population has been noted previously (Kotnik et al., 2023). This study is the first to describe the prevalence of genes associated with other cancer genes in individuals of the Slovenian population.

Of the miscellaneous gene list, pathogenic findings were most frequently discovered in the *HFE* gene (0.13%) associated with hemochromatosis, as male homozygotes with the p.C282Y variant have a high possibility of severe liver diseases (Grosse et al., 2018). A similar result (0.16%) has been previously reported in Slovenian blood donors (Cuk et al., 2007). This variant presents the most frequent *HFE* variant in the Slovenian hereditary hemochromatosis patients as well, and is present in 71.4% of that population (Stepec et al., 2008). On the other hand, pathogenic variants in this gene appear to be depleted in the Slovenian population compared to the general gnomAD population (OR 0.31; p = 0.01). The trend of decreasing of HFE variants from northwest to southeast Europe, with a higher prevalence in the populations that have a heavier Celtic or Viking component, has been reported previously (Distante et al., 2004). A higher proportion of homozygous individuals carrying this variant in gnomAD can be explained as North and West European populations represent the highest proportion of the gnomAD cohort (Karczewski et al., 2020).

Several studies uncovered the burden of pathogenic variants in different large unaffected populations and multi national cohorts, revealing that pathogenic variants in medically actionable genes of the ACMG list are present in the 2%–5% range worldwide. Amongst the large populations tested, pathogenic variants are present in 4.1% participants of the United Kingdom Biobank (Halldorsson et al., 2022), 3.4% of the US population (Savatt et al., 2025), 5.81% of the American paediatric population (Saeidian et al., 2024), 5.3% of the Chinese population (Huang et al., 2022), 2.26% of the European samples in the All of Us study (Venner et al., 2024), and 2.54% of the samples of the USA-based Emerge network (Gordon et al., 2020). There have also been some large scale European initiatives, such as 1+ Million genomes (Vicente, 2024) and the Genome of Europe (Uitterlinden, 2026), aiming to establish an European genomic infrastructure and a pan-European reference genome representing the rich ancestral and genetic diversity of the continent’s population. Still, such large scale studies have historically disproportionately prioritized larger populations, specifically those of Western and of Northern European ancestry, leaving the small Central European populations underrepresented (Fatumo et al., 2022). The task of addressing the population-specific burden of secondary findings in different small European populations is gradually being addressed through studies conducted at the national level (Grech et al., 2025). Present study is one of the first to evaluate the burden of clinically significant findings in a nation-based, Central European population.

The discovery of the prevalence of pathogenic variants in specific medically actionable genes is important since it may lead to discussions regarding population-specific, gnomically backed public health policies. For example, our work revealed that pathogenic variants in the Slovenian population are most frequently present in *LDLR*, *TTN* and the *BRCA* genes. The population screening in Slovenia is well established and efficiently functioning for both familial hypercholesterolemia and breast cancer (Sustar et al., 2022; Tomšič et al., 2025), whereas no population screening currently exists for cardiomyopathy. The findings of this study may therefore provide a basis for further discussion on whether more systematic approaches to identifying individuals at increased risk of cardiomyopathy should be considered in the Slovenian population. Our study is noteworthy for a large, population-based cohort, a focus on medically actionable genes of the ACMG version 3.3 gene list, and a careful manual review of all the included variants. Still, there are a few limitations. Firstly, our study population is based on a clinical sample with removed primary findings and not on the general population, therefore it does not present a true population based sample, but rather an approximation of one. Because of that, our sample could be enriched for secondary findings, family history, latent phenotypes or relatedness. Still, all of the 14.000 individuals included in our database have been seen by a genomic specialist, who has evaluated their clinical presentation and the genomic variants found in detail and established, whether the variants found present a primary finding or a secondary finding. For our control population we have used the gnomAD database, because it is the largest publically available database of unaffected individuals. Still, because the gnomAD database is enriched for individuals of Western and Northern European ancestry, using it as a control database for our Central European population may be a limitation, still, the comparison of the two revealed differences in the pathogenic variant burden in two genes in our study. Thirdly, our analysis and classification of the variants did not include functional analysis; therefore, we could not conclusively classify novel candidate pathogenic variants and VUS variant as either benign or pathogenic. Fourthly, our dataset was anonymized; therefore, no segregation analysis of the found variants was possible. Finally, our analysis includes single-nucleotide variants only, and some of the other types of variation, such as copy number variation, could present pathogenic findings. This study presents an important step in the description of the genetic landscape of the Slovenian population as well as an argument for careful interpretation of genomic data in our population, since one individual in 31 harbours a potentially medically actionable variant and, as such, presents a candidate for preventive treatment or early screening, of which he may be unaware. Our result of 3.21% of individuals in the Slovenian population harboring a pathogenic variant or a novel candidate likely pathogenic variant in a medically actionable gene provides an opportunity to discuss public health genomics in population cohorts to promote the evidence-based use of genomics for the prevention of hereditary disease and can inform future applications of genomics in public health (Green et al., 2019). In conclusion, we report a population burden of 2.73% of pathogenic variation and 0.48% of novel candidate likely pathogenic variation in genes, connected to medically actionable disease in a large, Slovenian population-based study.

## Data Availability

The original contributions presented in the study are publicly available. This data can be found in the European Variation Archive at EMBL-EBI under accession number PRJEB126801 at https://www.ebi.ac.uk/ena/browser/view/PRJEB126801.
